# Gut microbiota, short chain fatty acids, and obesity across the epidemiologic transition: the METS-Microbiome study protocol

**DOI:** 10.1186/s12889-018-5879-6

**Published:** 2018-08-06

**Authors:** Lara R. Dugas, Louise Lie, Jacob Plange-Rhule, Kweku Bedu-Addo, Pascal Bovet, Estelle V. Lambert, Terrence E. Forrester, Amy Luke, Jack A. Gilbert, Brian T. Layden

**Affiliations:** 1 Public Health Sciences, Stritch School of Medicine, Loyola University Chicago, 2160 S. 1st Avenue, Maywood, IL 60153 USA; 2 Department of Physiology, SMS, Kwame Nkrumah University of Science and Technology, Kumasi, Ghana; 3 Institute of Social & Preventive Medicine, Lausanne University Hospital, Lausanne, Switzerland; 4 Ministry of Health, Republic of Seychelles, Lausanne, Switzerland; 5 Research Unit for Exercise Science and Sports Medicine, University of Cape Town, Cape Town, South Africa; 6 Solutions for Developing Countries, University of the West Indies, Mona, Kingston Jamaica; 7 Microbiome Center, Department of Surgery, University of Chicago, Chicago, IL USA; 8 Division of Endocrinology, Diabetes, and Metabolism, University of Illinois at Chicago, Chicago, IL USA; 9 Jesse Brown Veterans Affairs Medical Center, Chicago, IL USA

**Keywords:** Epidemiologic transition, Gut microbiota, Short chain fatty acids, Obesity

## Abstract

**Background:**

While some of the variance observed in adiposity and weight change within populations can be accounted for by traditional risk factors, a new factor, the gut microbiota, has recently been associated with obesity. However, the causal mechanisms through which the gut microbiota and its metabolites, short chain fatty acids (SCFAs) influence obesity are unknown, as are the individual obesogenic effects of the individual SCFAs (butyrate, acetate and propionate). This study, METS-Microbiome, proposes to examine the influence of novel risk factors, the gut microbiota and SCFAs, on obesity, adiposity and weight change in an international established cohort spanning the epidemiologic transition.

**Methods:**

The parent study; Modeling the Epidemiologic Transition Study (METS) is a well-established and ongoing prospective cohort study designed to assess the association between body composition, physical activity, and relative weight, weight gain and cardiometabolic disease risk in five diverse population-based samples in 2500 people of African descent. The cohort has been prospectively followed since 2009. Annual measures of obesity risk factors, including body composition, objectively measured physical activity and dietary intake, components which vary across the spectrum of social and economic development. In our new study; METS-Microbiome, in addition to continuing yearly measures of obesity risk, we will also measure gut microbiota and stool SCFAs in all contactable participants, and follow participants for a further 3 years, thus providing one of the largest gut microbiota population-based studies to date.

**Discussion:**

This new study capitalizes upon an existing, extensively well described cohort of adults of African-origin, with significant variability as a result of the widespread geographic distributions, and therefore variation in the environmental covariate exposures. The METS-Microbiome study will substantially advance the understanding of the role gut microbiota and SCFAs play in the development of obesity and provide novel obesity therapeutic targets targeting SCFAs producing features of the gut microbiota.

**Trial registration:**

Registered NCT03378765 Date first posted: December 20, 2017.

## Background

Obesity is a complex condition with a multi-faceted etiology. To date, while some of the variance observed in obesity within populations can be accounted for by traditional risk factors such as total energy expenditure, physical activity (PA) level, dietary intake, genetics, socioeconomic status or education level [1–6], a new factor, the gut microbiota have also been recently implicated in obesity [7–9]. This study, METS-Microbiome, proposes to examine the influence of the gut microbiota, and its metabolites of carbohydrate digestion, short chain fatty acids (SCFAs), on obesity, and weight change.

The gut microbes are responsible for breaking down non-digestible dietary nutrients, such as pectin, cellulose, and resistant starches. Fermentation of these nutrients in the distal gut results in the production of SCFAs, mainly butyrate, propionate and acetate. Each of these is absorbed by the human gut and contributes approximately 200 kcal/day to total body energy expenditure [10]. SCFAs are a key energy source for the intestinal epithelium and liver [11], and consequently affect many metabolically important processes including hepatic gluconeogensis and lipogenesis [12, 13], gut barrier function [14, 15], gut motility [16] and immune responses [17, 18]. Importantly, digestion of resistant starches, with associated increases in fecal SCFA concentrations, has been shown to increase satiety, and is associated with improvements in blood glucose and cholesterol [19, 20].

There are approximately 39 trillion bacterial cells in the human microbiota in an average-sized man, whereas there are 30 trillion human cells in the body [21]. Currently bacteria can be classified in 29 phyla and 5569 taxa, along the List of Prokaryotic names with Standing in Nomenclature [22]. Three main phyla colonize the gut; Firmicutes, Bacteroidetes and less abundantly by the Actinobacteria. In humans, studies indicate an increase in the Firmicutes and a decrease in the Bacteroidetes phyla to be associated with obesity [23, 24], although not all studies have observed this [25, 26]. In one of the earliest human studies, Ley et al. [23] compared the gut microbiota of 12 obese individuals, following two different low calorie diets over the period of 1 year and found that at baseline, obesity was associated with fewer Bacteroidetes (*p* < 0.001). However, with subsequent weight loss, there were increases in the Bacteroidetes, concomitant with decreases in the Firmicutes phyla, and thus an increased Bacteroidetes/Firmicutes ratio, irrespective of diet assignment. Ferrer et al. [24] confirmed these findings comparing the gut microbiota in lean and obese individuals. While many postulated mechanisms of how the gut microbiota contributes to obesity have been suggested [9, 27–30], the focus of this study will be on addressing the relationship between the gut microbiota and SCFAs.

The gut microbiota in and of itself appears to be influenced by many external factors in the host’s environment [31], thus when investigating this microbial ecosystem, other influencing external factors must be considered [32, 33]. Previous studies [34, 35], however, are limited by contradictory findings [36], small sample sizes [37–42], imprecise measurements of obesity [43, 44], and lack of detailed dietary and other environmental exposures/mediators [38, 41, 45]. The parent study; Modeling the Epidemiologic Transition Study (NIH R01-DK080763) is a well-established and ongoing prospective cohort study designed to assess the association between body composition, PA, and relative weight, weight gain and cardiometabolic disease risk in five diverse population-based samples of African descent. The five international research sites include Ghana, South Africa, Jamaica, the Seychelles, and the US. The new study, METS-Microbiome (NIH R01-DK111848), is therefore well suited to examine the role a host’s local environment has in the associations between the gut microbiota, SCFAs and adiposity. Indeed, each of the 5 METS sites has been well characterized for their own unique environmental and dietary/lifestyle sources of exposure [2, 46–55]. Notably, sites differ according to levels of adiposity (measured using dual x-ray absorptiometry), ranging from 28% in Ghana, up to 39% in the USA, dietary composition (averaged from two 24 h recall), where %diet from carbohydrate ranges 46% in the USA up to 66% in Ghana, as well as differing levels of physical activity (PA, objective activity monitoring).

Using our epidemiologic model for studying the associations between the gut microbiota, SCFAs and the development of obesity, we can explore the interplay of these factors independently and collectively (e.g. dietary habits, daily PA, socio-economic status, public health policy as well as access to health care). In fact, this model has been key to our understanding of obesity and also other chronic diseases in the modern world [4, 54, 56–69]. However, the human gut microbiota, SCFAs and its implications for the obesity epidemic, is only now being considered in detail [37–39, 45]. Interestingly, and to the best of our knowledge, the gut microbiota and SCFAs have not been considered in relationship to the epidemiologic transition model. By exploring these variables through the epidemiologic transition model, we will be able to capture these interactions, and provide novel insight into the obesity epidemic as well as explore innovative therapeutic targets. In fact, we have just published a review justifying the use of this epidemiologic model to unpack the role of the gut microbiota [70].

In summary, the significance of METS-Microbiome is that it may clarify the relationships between gut microbiota, SCFAs and obesity across diverse environments. Also, it may provide novel therapeutic targets, which might be considered as part of the multi-faceted obesity treatment approach. Specifically, if SCFAs mediate the relationship between gut microbiota and obesity, targeting them through either dietary, probiotics, or pharmaceutical intervention may provide additional therapeutic tools in treatment of obesity.

### METS-microbiome study hypotheses and aims

The METS-Microbiome study was designed to test three hypotheses associated with the relationship between novel risk factors, the gut microbiome and SCFAs, on obesity, adiposity and weight change. We hypothesized that: 1) there exists a shared gut microbiota and SCFAs production are etiological factors in obesity across populations, 2) gut microbiota and SCFAs factors cross-sectionally associated with adiposity will be predictive of longitudinal changes in adiposity, 3) The relationship between gut microbiota and SCFAs production is both shared, yet also reliant on local environmental stimuli.

## Methods

### Design and settings

The parent study; METS, is a well-established and ongoing prospective cohort study designed to assess the association between body composition, PA, and relative weight, weight gain and cardiometabolic disease risk in five diverse population-based samples of African descent (NIH R01-DK080763). A description of the METS protocol for centralized field staff training, data collection, measurement and laboratory procedures has been published [71]. To date, 26 METS-related manuscripts have been published or in press [2, 18, 46–55, 68, 72–74].

In the original METS study, 2,506 (*N* = 2,506) young adults, age 25–45 years, were enrolled at baseline between January 2010 and September 2011 with 500 participants (~ 50% male) from each of five sites: rural Ghana (Kumasi), peri-urban Republic of South Africa (Cape Town), island nation Seychelles (Mahé), urban Jamaica (Kingston) and suburban Chicago (Maywood, IL) in the United States (USA). These five sites were chosen to represent the spectrum of the ‘epidemiologic transition’ with Ghana and the USA representing the two extremes. Populations sampled represent a range of social and economic development as defined by the United Nations Human Development Index (HDI)(UN [75]). Baseline characteristics of the cohort, are presented by HDI site ranking in Table 1. As a result of the cohort design, average baseline BMI varied widely across sites and obesity (BMI ≥ 30) prevalence ranged from 1.4% (Ghanaian men) to 63.8% (USA women).

**Table 1:** Baseline Characteristics of the Original METS Cohort (2506) by Site^a^ (mean ± SD, %)

	Ghana (*n* = 500)	South Africa (*n* = 504)	Jamaica (*n* = 500)	Seychelles (*n* = 500)	USA (*n* = 502)
Age (y)	34.3 ± 6.7	33.4 ± 5.8	34.4 ± 6.1	36.1 ± 5.6	35.3 ± 6.3
BMI (kg/m^2^)	24.1 ± 4.6	27.5 ± 8.1	26.5 ± 6.4	27.1 ± 5.7	31.9 ± 8.5
Fat Mass (kg)	18.5 ± 9.2	29.0 ± 16.0	24.7 ± 13.4	25.8 ± 11.1	37.7 ± 18.3
Body Fat (%)	28.3 ± 10.3	36.8 ± 11.4	31.0 ± 11.4	33.1 ± 9.2	39.1 ± 11.0
Plasma Glucose (mg/dL)	99.7 ± 12.1	84.0 ± 23.0	93.1 ± 9.4	100.7 ± 29.1	100.1 ± 34.8
% Carbohydrate (%Energy)	65.8 ± 10.4	54.9 ± 11.8	58.5 ± 8.5	51.2 ± 9.3	45.9 ± 9.4
% Protein (%Energy)	11.9 ± 4.0	16.6 ± 4.8	14.8 ± 4.1	18.4 ± 4.7	15.5 ± 4.1
% Fat (%Energy)	21.7 ± 9.2	26.4 ± 9.3	25.6 ± 6.6	28.4 ± 7.6	36.6 ± 7.0
Fiber (g/day)	25.0 ± 9.7	8.7 ± 4.0	16.5 ± 8.4	13.5 ± 6.7	14.2 ± 7.1
Soluble Fiber (g/day)	6.0 ± 2.8	2.9 ± 1.4	4.9 ± 2.7	3.9 ± 2.1	4.6 ± 2.4
Insoluble Fiber (g/day)	18.9 ± 7.5	5.8 ± 2.9	11.6 ± 6.1	9.6 ± 5.1	9.5 ± 5.38
Obesity (%)	9.8	32.1	27.6	26.4	52.4
Diabetes (%)	1.0	2.4	0.2	3.4	9.6

^a^Sites are presented according to their level of Epidemiologic Transition, using Human Development Index rankings. Diabetes, random glucose > 140 mg/dL or currently taking diabetes medication

For the new study; METS-Microbiome (R01-DK111848), data and biological samples collected during subsequent years of follow-up examinations will be utilized for year 8–10 follow up (2018–2021). As indicated, a total of 2506 participants were recruited at baseline (2010–2011). At present, across all sites, we have approximately 65% retention of the original cohort. Recruitment and replacement of participants lost to follow-up has begun in the sites.

### Ethics approval

The protocol for METS-Microbiome was approved by the Institutional Review Board of Loyola University Chicago, IL, USA; the Committee on Human Research Publication and Ethics of Kwame Nkrumah University of Science and Technology, Kumasi, Ghana; the Research Ethics Committee of the University of Cape Town, South Africa; the Board for Ethics and Clinical Research of the University of Lausanne, Switzerland; the Health Research and Ethic Committee of the Ministry of Health of Seychelles, and the Ethics Committee of the University of the West Indies, Kingston, Jamaica. The study strictly adheres to the principles and protocols from the Declaration of Helsinki. The study was registered prospectively with the U.S. National Library of Medicine ClinicalTrials.gov website on December 20, 2017, and began recruiting in January 2018. The study was assigned the following ClinicalTrials.gov identifier: NCT03378765, and is funded by the National Institutes of Health R01 mechanism (R01-DK111848).

### Biological samples and measurements

For METS-Microbiome, 3 years of data will be collected, coinciding with years 8–10 of the original METS study. Project coordinators for each field site were trained and certified in all measurement protocols by coordinating center staff; the measurements included in the METS-Microbiome study are summarized in Table 2**.** In brief, anthropometrics including weight, height, waist and hip circumferences have been collected using standardized methods and the same equipment [68]. Blood pressure is measured in triplicate at two-time points during each examination using an automatic digital monitor (model HEM-747Ic, Omron Healthcare, Bannockburn, IL USA). Body composition is assessed in all participants at each examination using bioelectrical impedance analysis and study-specific Eqs. (55). Fasting plasma glucose will be measured; insulin, leptin and adiponectin will be measured in fasting plasma samples using radioimmunoassay kits (Linco Research, Inc., St. Charles, MO). Spot urines will be collected at baseline and assayed for urinary albumin and creatinine levels. Unused whole blood, plasma, serum and urine samples are stored at -80C for use in future analyses. Fecal samples will be analyzed for both gut microbiota and SCFAs, in all participants from Year 8–10 samples.

**Table 2:** Proposed Study Measures

	Baseline	Follow-Up Examinations		
		Years 1–5	Year 8	Years 9–10
METS				
Physical Activity				
Accelerometer	X	X	X	X
Questionnaire	X		X	X
Body Composition				
BIA	X	X	X	X
Isotope Dilution	X	X		
DXA^a^		X		
Dietary Intake	X		X	
Anthropometrics	X	X	X	X
Serum & Urine Measures^b^	X	X	X	X
Health history, Demographics, etc.	X	X	X	X
METS-Microbiome				
Blood SCFAs	X		X	X
OGTT			X	
Gut Microbiota			X	X

^a^DXA for body composition and bone mineral density measures not available only in Seychelles

^b^Serum measures include fasting glucose, insulin, adiponectin, leptin, lipids, CRP, cystatin C at baseline and glucose, adiponectin and leptin at follow-up examinations

### Year 8–10 follow-up examination

All participants, including the original cohort and the new recruits, will undergo the Year 8–10 examination, and as described in the original METS protocol manuscript [49]. Anthropometrics, blood pressure, body composition by BIA, physical activity by accelerometry (Actical; Philips Respironics, Bend OR), and health and medication history by questionnaire will be collected. Extensive information is collected at each examination regarding self-reported health history, focusing on changes to health status since prior visits. Data on drinking, smoking and drug use, prescribed, over-the-counter and illicit, are collected at each examination, along with measures of socioeconomic status, education, employment status and history [76], and physical activity by questionnaire [77]. Site-specific food frequencies will be administered to participants by trained study staff. All participants will undergo an oral glucose tolerance test (OGTT) to assess glucose tolerance and insulin secretion and sensitivity using Minimal Model analysis Participants will be asked to provide a fecal sample using a standard collection kit (EasySampler stool collection kit, Alpco, NH).

### Oral glucose tolerance test

Participants will be instructed to fast overnight and refrain from exercise prior to the test. A standard 75-g OGTT will be performed and blood samples will be drawn at 0, 30, 60, 120 min for subsequent determination of plasma glucose, and serum insulin and C-peptide concentrations. Impaired glucose metabolism will be defined using standard criteria as suggested by the ADA [78] in accordance with the IDF [79] and WHO [80].

#### Measurement of short chain fatty acids in stool

SCFAs (acetate, propionate, butyrate, formic acid, and isovaleric acid) will be isolated from 10 mg fecal aliquots, and measured using gas chromatography-mass spectrometry (GC/MS), according to the methods outlined in Moreau et al. [81] and Richardson et al. [82]. Briefly, in an aliquot of 10 mg fecal matter sample (with total protein content analysis by Bradford assay for normalization), add 2-ethylbutyrate internal standard in 0.5 ml water and 0.1 ml concentrated hydrochloric acid, shake 30 min with 1 ml MTBE including methylbutyrate internal standard. Decant MTBE phase, dry over sodium sulfate, derivatize with MTBSTFA at 80 °C for 30 min, inject 1 μl onto a 30 m 0.25 mm, 0.25 um DB5 duraguard column in a GC/MS with temperature gradient 50–290 °C, scanning 50–550 Da. Spiked recoveries in fecal matter range from 65 to 110% for formic acid to valerate. All short chain fatty acids had better than 7% within- and between-batch reproducibility and quantification limits < 10 pmol injected onto the column. Fecal samples will be centrally stored at -80°C at Loyola University Chicago after shipment from the field sites.

#### Measurement of gut microbiota

##### DNA extraction, multiplex 16S allele PCR and sequencing

We will quantify microbiome features from amplicon data using existing pipelines [83] to identify strain-level taxonomic markers for all samples. Microbial DNA will be extracted using the PowerSoil-htp 96-well Soil DNA Isolation Kit (MoBio). The 16S rRNA V4 regions will be PCR-amplified and sequenced using the Illumina HiSeq 2500 platform to generate ~ 100,000,250 bp paired-end reads per sample [84]. All amplicon sequencing data will be quality filtered and de-multiplexed and then subjected to de novo operational taxonomic unit (OTUs) picking, and subOTU characterization using DeBlur [85]), via the QIIME platform.

### Bioinformatics data analysis

We will perform a Microbiome Wide Association Study (MWAS; [86]) to determine whether fecal microbial biomarkers are predictive of participant variables. Microbial 16S rRNA diversity will be summarized using Chao1 estimator and Shannon index, and the relative proportions of specific taxa. Significant relationships will be tested using generalized linear modeling. UniFrac distances (between-sample beta-diversity), microbial 16S rRNA diversity (alpha diversity, including evenness) will be correlated against the obesity and SCFA variables using multivariate methods such as principal coordinate analysis (PCoA), Analysis of the Composition of Microbiomes (ANCOM; [87]), and permutational multivariate analysis of variance (PERMANOVA). We will also employ correlative network modeling, including correction of multiple testing, to determine if the network associations (based on relative abundance correlation) differs with population, obesity and SCFA concentrations; such differences in node-level topological features of the network can help with interpreting ecological variability in the stability of each microbiome [88, 89]. We will characterize the modularity of these networks using a random walk approach and link these community structures to sample type via random forest modeling and multinomial logistic regression. Relative abundance of bacterial species will be characterized using sub-operational taxonomic unit level [85] . Based on DESeq2 results [90], logistic models will be fit using patient characteristics and SCFA concentrations as dependent variable and microbiome data as independent variables. Variable selection will be integrated to avoid over-fitting. Classification performance will be evaluated using ROC curve and the 0.632+ bootstrap method [91]. Random Forests will also be applied to determine whether the microbiome is predictive of participant variables [92].

### Shotgun sequencing

We will perform shotgun metagenomic sequencing (20 million reads per sample) to characterize the functional metabolic pathways that may be enriched or depleted in different populations, obesity groupings, or SCFA concentrations. Libraries will be generated using 1 ng of input DNA with the Nextera XT protocol (Illumina), and sequenced on the Illumina HiSeq platform (150 bp × 2, 10 samples per lane, Insert size range = 300 bp to 1200 bp). Raw metagenome reads will be quality trimmed using the nesoni pipeline [93]. Phylogeny will be assigned to reads using MetaPhlAn [94]. Reads will be assembled using IDBA_UD [95], and population genomes will be binned using MetaBAT [96]. Single copy marker gene based copy number variation analysis [97] will be used to estimate completion and intra-species contamination in each genome. Reconstructed genomes will be annotated using RAST [98]. These genomes will be cross-referenced against the 16S rRNA amplicon results and comparative genomes between different strains will be regressed against participant variables (e.g. BMI, SCFAs, etc). Functional genes and metabolic pathways will be identified and statistically analyzed using HUMAN [99], and Hidden Markov Models [100] with DIAMOND [101] and the KEGG database [102]. Relative abundance will be assign to each KEGG Ortholog detected. Finally, we will apply Predicted Relative Metabolomic Turnover [103] to produce a predicted metabolite profile based on pathway reconstruction. The relative proportions of specific taxa, genes, or predicted metabolites associated with outcomes will be tested by regression analysis and generalized linear modeling. UniFrac distances (between-sample diversity) will be correlated with participant variables in principal coordinate analysis (PCoA), permutational multivariate analysis of variance (PERMANOVA).

### Data management

Loyola University Chicago is the coordinating center for the current study. All data forms, questionnaires and dietary recall instruments are scanned and, along with electronic Actical data files, sent via secure transfer to the data manager at the coordinating center. Scanned forms are coded and double data entered by experienced, trained study staff. A series of logic checks are then performed and, when outliers are encountered, discrepancies are followed up with staff at the appropriate field site.

## Statistical considerations

### Overall approach and preliminary analysis

To ensure optimal model selection and protect against model overfitting, cross-validation techniques will be used to develop the models. The entire dataset will be randomly split into a training (60% of data) dataset and a test dataset (40%). Data will be divided using block randomization by site and gender to ensure equal contributions from the five sites. Models from each statistical approach will be fit using the training dataset and associations/predictions will be estimated on the validation set. Optimization of models and variable selection will involve a combination of stepwise selection and AIC criterion via 5-fold cross-validation [104]. The average validation error will also be used to assess model performance among the different statistical approaches and to choose the best approach for modeling this data. Univariate and bivariate summary statistics and distributional plots will be examined for all variables and appropriate transformations considered. Outliers will be identified using the “letter value” procedure which displays mild and severe outliers at the tails of the distribution [105, 106]. Associations between variables of interest will initially be explored with use of smooth scatter plots for continuous variables and cross-tabulations for discrete variables. Participant characteristics and baseline SCFAs values will be summarized by sex: overall and by site. Additionally, SCFAs concentrations will be examined by participant characteristics representing demographic and lifestyle factors that may be associated with exposure and outcomes of interest. Univariable comparisons will be examined via Pearson’s chi-square tests, Pearson’s correlation coefficients and Student’s t-tests, as appropriate. To account for potential differences in SCFAs levels by site, all modeling will adjust for site (in addition to age and gender) and, when feasible, modeling will be conducted within site to determine site-specific effects. All analyses will be performed using SAS version 9.4 (SAS Institutes, Cary, NC) and computing environment R (R Development Core Team, 2005).

### Covariates of interest

Based on biological considerations, it will be important to consider the following variables, among others, as covariates of interest in our analyses: age, sex, site, BMI, family or previous history of diabetes or currently taking medications for diabetes or hypertension, blood pressure, nutritional status indicators (e.g., dietary nutrient and specific food intakes), smoking, alcohol use, education, occupation and employment status, marital status, parity (females) and physical activity.

## Discussion

This study capitalizes upon an existing, extensively well described cohort of adults of African-origin initiated in 2009, with significant variability as a result of the widespread geographic distributions, and therefore variation in the environmental covariate exposures. The METS-Microbiome study will substantially advance the understanding of the role gut microbiota and SCFAs play in the development of obesity and provide novel obesity therapeutic targets targeting SCFAs producing features of the gut microbiota. Specifically Studying unique populations as they span the epidemiologic transition, allows us to investigate several risk factors simultaneously, including environmental co-variates, (e.g. local diet/PA), which have been shown to impact both gut microbiota and SCFAs. In addition, continuing follow-up in a previously established, longitudinal cohort of African-origin adults, and considered high-risk for the development of obesity and metabolic disorders will allow us to capitalize on identifying causal factors. Notably, we can leverage up to 10 years of detailed phenotype information already collected in the parent study, METS, including yearly weight change, to model the contribution of these on the gut microbiota and SCFAs. METS-Microbiome will utilize observational measures include gold-standard techniques such as DXA body composition and objective PA monitoring, in a large, well characterized population cohort and thus address potential confounding such as diet/PA, as well local environmental stimuli.

The investigative team, with its geographic diversity, existing data, established partnerships, and multidisciplinary expertise, is uniquely positioned to conduct this type of research. With this study, we have enriched the existing METS investigative team of epidemiologists, biostatisticians, nutritionists and exercise physiologists with expertise in endocrinology, microbiology, metabolomics and bioinformatics. This transdisciplinary approach will allow for careful and thorough examination of every phase of the study.

In conclusion, the proposed study will explore the unknown causal mechanisms though which SCFAs mediate the relationship between the gut microbiota and adiposity. As a result of the large and diverse cohort, as well as the comprehensive study design, METS-Microbiome has the potential to uncover several new potential mechanisms involved with development of obesity across populations spanning the epidemiologic transition.

## Abbreviations

ADA: American diabetes sssociation
ANCOM: Analysis of the composition of microbiomes
BIA: Bioelectric impedance analysis
BMI: Body mass index
GC/MS: Gas chromatography-mass spectrometry
HDI: Human development index
IDF: International diabetes federation
METS: Modeling the epidemiologic transition
OGTT: Oral glucose tolerance test
OTUs: Operational taxonomic unit
PA: Physical activity
PCoA: Principal coordinate analysis
PERMANOVA: Permutational multivariate analysis of variance
SCFAs: Short chain fatty acids
USA: United States of America
WHO: World Health Organization
